# Clinical pilot study: efficacy of triple antibiotic therapy in *Blastocystis* positive irritable bowel syndrome patients

**DOI:** 10.1186/s13099-014-0034-0

**Published:** 2014-08-20

**Authors:** Robyn Nagel, Helle Bielefeldt-Ohmann, Rebecca Traub

**Affiliations:** 1 School of Veterinary Science, The University of Queensland, Gatton, Queensland, Australia; 2 Australian Infectious Diseases Research Centre, The University of Queensland, St. Lucia, Australia; 3 Faculty of Veterinary Science, The University of Melbourne, Parkville 3052, Victoria, Australia; 4 Toowoomba Gastroenterology Clinic, Suite 105 Medici Medical Centre, 15 Scott Street, Toowoomba 4350, Queensland, Australia

**Keywords:** Blastocystis, Therapy, Irritable bowel syndrome, PCR

## Abstract

**Background:**

*Blastocystis* species are common human enteric parasites. Carriage has been linked to Irritable Bowel Syndrome (IBS). Treatment of *Blastocystis spp*. with antimicrobials is problematic and insensitive diagnostic methods and re-infection complicate assessment of eradication. We investigated whether triple antibiotic therapy comprising diloxanide furoate, trimethoprim/sulfamethoxazole and secnidazole (TAB) given to diarrhoea-predominant IBS (D-IBS) patients positive for *Blastocystis* would achieve eradication.

**Methods:**

In a longitudinal, prospective case study 10 D-IBS *Blastocystis*-positive patients took 14 days of diloxanide furoate 500 mg thrice daily, trimethoprim/sulfamethoxazole 160/80 mg twice daily and secnidazole 400 mg thrice daily. Faecal specimens were collected at baseline, day 15 and 4 weeks after completion of TAB. Specimens were analysed using faecal smear, culture and polymerase chain reaction (PCR) of the 16 SSU rRNA. Patients kept a concurrent clinical diary.

**Results:**

Six (60%) patients cleared *Blastocystis spp.* after TAB, including three who had failed previous therapy. Subtypes detected were ST3 (60%), ST4 (40%), ST1 (20%) and ST7, 8 (10%); four patients had mixed ST infections. Serum immunoglobulin A (IgA) levels were low in 40% of patients. Higher rates of *Blastocystis* clearance were observed in patients symptomatic for less than a year (Mann–Whitney, p = 0.032, 95% confidence) with no associations found with age, previous antibiotic therapy, faecal parasite load, ST, IgA level or clinical improvement.

**Conclusions:**

Clearance of *Blastocystis* spp. was achieved with TAB in 60% of D-IBS patients, an improvement over conventional monotherapy. Higher clearance rates are needed to facilitate investigation of the relevance of this parasite in clinically heterogenous IBS.

## Background

*Blastocystis spp*.are unicellular, anaerobic enteric parasites found world wide in almost all species of animals [1]. The cystic form survives for days in the environment and likely transmits infection by the faecal-oral route either by direct contact or via contaminated water supplies. *Blastocystis* spp. are the most common parasite found in human faeces and carriage has been reported to be associated with Irritable Bowel Syndrome (IBS)–like symptoms [1]–[3]. IBS is a chronic heterogenous disorder present in up to 10% of the global population characterised by a symptom complex of abdominal pain and variable bowel habit in the absence of gross morphological, histological or inflammatory diagnostic markers [4]. Although IBS does not increase mortality the disease impairs quality of life and costs billions of dollars annually in direct and indirect health care costs [5]. Genetic, environmental, gender and psychological factors are known to be important in the development of the disease but the pathophysiology is unknown. As many as 10% of cases of IBS are recognised to develop after a bout of acute infectious gastroenteritis and this has focused research on microbial interactions with the luminal gut mucosa [6].

Faecal microbiota profiles are reported to be significantly different in IBS patients, most notably in the diarrhoea predominant IBS (D-IBS) subgroup [7]. IBS patients have diminished faecal bacterial diversity with temporal instability, fewer aerobic bacteria and decreased *Bifidobacterium* spp./*Enterobacter* spp. ratio [7],[8]. Altered intestinal motility and abdominal hypersensitivity are typically seen in IBS, and many researchers have reported subtle alterations of the enteric immune and nervous system of IBS patients [9]. Increased numbers of mast cells secreting serotonin that are closely aligned to mucosal dendritic cells are consistently observed in the gastrointestinal mucosa of patients with IBS [10]. Changes in cytokine secretion are reported not only with many luminal enteric microbial infections but increased levels of pro-inflammatory cytokines (interleukin (IL)-6, IL-8, and tumour necrosis factor-α (TNF-α)) have been reported in the plasma and peripheral blood mononuclear cells of IBS patients [6]. Many of these cytokines increase epithelial permeability by disrupting the mucosal tight junctions [11]. Proteases are also reported to be elevated in the faeces of IBS patients [12],[13] although it is not clear if these proteases derive from enteric microorganisms, human intestinal cells or both. *Blastocystis* organisms have been shown to activate IL-8 gene expression in human colonic epithelial cells *in vitro*[14], increase epithelial permeability [15] and degrade intestinal Immunoglobulin A (IgA) [16], mediated possibly by action of cysteine proteases [17] reported to be present on the cell surface [18].

The prevalence of *Blastocystis* carriage is increased in all IBS patients but highest in D-IBS patients (73% compared to 27% in healthy control patients) [19], suggesting that this subgroup may provide a more homogenous IBS patient group to study. Seventeen different subtypes (ST) of *Blastocystis* spp. have been described to date [20] revealing moderate host ST specificity and regional ST variation in humans. Early reports that particular subtypes of *Blastocystis* may be associated with clinical pathogenicity in humans have not been confirmed [1]. Treatment of *Blastocystis* spp. with antimicrobials is problematic and insensitive diagnostic methods and possible re-infection complicates assessment of successful eradication [21],[22]. Monotherapy with metronidazole is the most commonly recommended drug and published eradication rates vary from 0-100% [21]. If *Blastocystis* spp. could be predictably successfully eradicated in symptomatic patients then it may be possible to more easily evaluate whether infection is associated with clinical symptoms. In this pilot study we treated ten patients with diarrhoea-predominant IBS (IBS-D) who were positive for *Blastocystis* carriage with triple antibiotic therapy (diloxanide furoate, trimethoprim-sulfamethoxazole, secnidazole) and assessed clinical status, ST and clearance rates.

## Materials and methods

### Study outline

This prospective, longitudinal study was conducted in a rural specialist outpatient clinic (Toowoomba Gastroenterology Clinic). Patients presenting with chronic diarrhea were clinically assessed and patients who were subsequently diagnosed with diarrhoea-predominant IBS (IBS-D) [4] and were positive for *Blastocystis* carriage were invited to participate in the study. Faecal specimens were collected from study participants at baseline, after two weeks of triple antibiotic therapy and six weeks after completion of antibiotics.

### Inclusion protocol

Adult patients presenting to the clinic with chronic diarrhoea from 1/8/11 to 20/02/14 were assessed clinically. Blood tests, including full blood count, serum calcium, thyroid function tests, serum immunoglobulin A (IgA) and coeliac antibody tests were performed. Serum IgA testing was performed using a nephelometry assay using a BNII device (Siemens, Germany). The reference range for a healthy population provided by the commercial laboratory was 1.24-4.16 g/L. Selective IgA deficiency is defined as <50% of the lower range of normal (0.61 g/L). Faecal microscopy for ova, cysts and parasites (including *Dientamoeba fragilis* trophozoites), culture (for bacterial pathogens including *Salmonella* sp, *Shigella* sp, Vibrio *cholerae*, *Campylobacter* sp, *Aeromonas* sp) and PCR analysis for *Giardia duodenalis*, *Clostridium difficile* and *Entamoebae histolytica* (and a 3-day culture for hookworm) were performed by a commercial pathology laboratory to exclude known pathogens and initially detect the presence of *Blastocystis* spp. All patients proceeded to an upper and lower endoscopy that included gastric antral, duodenal, ileal and colonic biopsies. Ten consecutive, eligible symptomatic patients positive for *Blastocystis* carriage who had no other cause for symptoms identified and who fulfilled the Rome criteria [4] for D-IBS, namely symptoms of chronic abdominal pain, occurring frequently over the last 3 months, associated with defecation and the passage of predominantly loose stool, were enrolled in the study. All patients who were invited to participate consented to enrolment and completed the study.

### Exclusion protocol

Only non-pregnant adults between 18 and 75 years of age were recruited for the study. Patients with a known allergy to sulphur drugs or patients with significant systemic diseases or co-morbidities were excluded.

### Study protocol

One baseline faecal specimen was collected from patients prior to 14 days therapy with diloxanide furoate 500 mg three times daily, trimethoprim/sulfamethoxazole 160/800 mg twice daily and secnidazole 400 mg three times daily. Two further faecal samples were taken, the first within 48 hours of ceasing antibiotics and another 4 weeks after ceasing antibiotics. Patients were reviewed clinically at the start of the study, after completion of antibiotics and 6 weeks later. All patients kept a diary which recorded the number of daily bowel movements (number per day), consistency of the stool (scored: 1 = very hard, 2 = hard, 3 = formed, 4 = loose, 5 = watery) and general well being (scored 1–10; from poor to excellent). Routine electrolytes, liver function tests and a full blood count were repeated 4 weeks after ceasing the antibiotics.

### Diagnostic methods

All samples were run in parallel for the presence of *Blastocystis* spp. using a simple unstained wet faecal smear, xenic *in vitro* culture (XIVC) and PCR (confirmed as *Blastocystis* spp. using DNA sequencing). A patient was considered to be positive if any one of the tests was positive.

### Parasitological methods

Fresh unpreserved faecal specimens from patients were examined as a fresh faecal smear under 40× magnification by light microscopy. The load of *Blastocystis* organisms was classified as light, medium or heavy (<5, 5–10 and >10 organisms per high powered field (O/HPF). Additionally 100 mg of fresh stool was inoculated in Jones’ culture medium [23] and incubated at 37°C for 48 hours. Cultures were examined for the presence of *Blastocystis* spp. by light microscopy.

### Molecular methods

DNA was extracted from the unpreserved faecal samples and pelleted cultures of *Blastocystis* using the QIAamp DNA Stool Mini Kit (Qiagen, Hilden, Germany) according to the manufacturers instructions, except that following the addition of lysis buffer, the faecal suspension was subjected to three freeze-thaw cycles in liquid nitrogen and a 95°C water bath respectively.

The genomic DNA from stool and cultured *Blastocystis* specimens from all patients was subjected to PCR analyses. All samples were analysed using the Wong protocol [24] that amplified a 1000 bp of the (SSU) rRNA gene of ST 1–17 of *Blastocystis* spp. All positive PCR products were subjected to DNA sequencing and phylogenetic analysis to identify the particular ST.

All *Blastocystis* spp. positive samples were also analysed separately with three specific ST primers (ST1, 3, 4) in order to detect mixed infections. The ST1 specific protocol was developed by Dr CR Stensvold and the ST3 and ST4 protocols were developed in-house. These primers amplify highly ST-specific smaller fragments of the (SSU) rRNA gene and obviate the need for PCR product sequencing. The ST specific primers 1 and 4 were specific for ST when tested against Netski (ST1) and WR1 (ST4) axenic cultures. The STS primer 3 demonstrated mild cross reactivity with ST1 and ST4 and if disparity was found between the Wong primer results and the STS primer result the PCR product was sequenced. The ST specific primers sequences are as follows:

### ST1: Forward:TAATACATGAGAAAGTCCTCTGG

Reverse: CTCAATCCAATTGCAAGAC

Annealing temp: 57°C; amplicon size: 336 bp

ST3: Forward: GTAGTTGTGTAATGAATACATTCG

Reverse: GTCCCTTTTAATCTTATTCTCATG

Annealing temp: 62°C; amplicon size: 250 bp

ST4: Forward: GTGATCTTCGGATGACGTGAATC

Reverse: CAGAATTTCACCTCTGACTATTG

### Annealing temp: 60°C amplicon size 290 bp

PCR products were run on gels of 1% agarose in 1 x sodium borate (SB)(0.4 gmNaOH/2.25 g Boric acid/1 L H_2_O) buffer at 200 V in a Biorad electrophoresis system and were purified using a Purelink PCR Purification Kit (Invitrogen, Grand Island, NY, USA) prior to sequencing.

Sequencing was done using an ABI 3130xl Genetic Analyzer (Applied Biosystems) using a Ready Reaction Kit Version 3.1 (PE Applied Biosystems, California, USA). Sequences were then edited and assembled using Finch TV v 1.4.0.

DNA sequences were aligned with 15 reference sequences of *Blastocystis* SSU rRNA genes sourced from GeneBank (NCBI) representing the established subtypes described from mammals and birds. Distance-based analyses were conducted on readable sequences using Kimura 2-parameter distance estimates and trees were constructed using the Neighbour Joining algorithm using Mega 3.1 [25] software. *Preoteromonas lacertae* (U37108) was used as an out-group. Bootstrap analyses were conducted using 1000 replicates.

### Ethics approval, statistics

The study was approved by the University of Queensland Medical Research Ethics Committee and registered with the Australian and New Zealand Clinical Trials registry (http://www.ANZCTR.org.au) ACTRN: 12611000918921. The results were analysed using the Mann–Whitney U test (Wizard® statistical program).

## Results

### Clinical characteristics of the study group

Male (1) and female (9) patients with an age range of 28–73 years (median age 49 years) participated in the study. The shortest symptom duration was 3 months and the longest 10 years and the presenting symptoms were chronic diarrhoea and abdominal pain (10/10). Six patients had received prior antibiotic therapy (including metronidazole, norfloxacillin, nitazoxanide) for *Blastocystis* infection that did not clear either the parasite in the faeces or their symptoms. The remaining patients were naïve to therapy. During the period of the study no patient had any organisms other than *Blastocystis* spp. noted on faecal testing. No patient demonstrated eosinophilia on the peripheral blood film. There were no abnormalities noted in the baseline electrolyte, liver function tests or full blood films blood tests. Five patients recorded a low baseline serum IgA level (<1.24 g/L).

### Antibiotic therapy and clinical follow up

All patients completed 14 days of the triple antibiotic therapy (TAB). No serious adverse effects were noted; four patients complained of nausea, two patients of headache and one developed oral thrush and bloating during the antibiotic therapy course. At four weeks post antibiotic therapy repeat electrolytes, liver function tests and full blood count testing remained normal in all patients

### Faecal smears, cultures and PCR results

Faecal smear results prior to the commencement of treatment demonstrated a parasite load of <5 O/HPF in five patients, 5–10 O/HPF in four patients and >10 O/HPF in one patient. Five patients had cleared the *Blastocystis* parasite from faeces on testing with faecal smear, XIVC and PCR at Day 15 and six patients had similarly cleared the organism at 6 weeks. All baseline samples positive to either microscopy or culture were positive on PCR testing using the Wong primers. Four patients had mixed ST infections that were confirmed with the ST-specific primers and subsequent sequencing if indicated. The *Blastocystis* ST’s detected were ST3 (60%), ST 4 (40%), ST 1 (20%) and ST7 and ST8 (10%). Day 15 and week six samples demonstrated good correlation between faecal smear/XIVC results and PCR testing (Table 1).

**Table 1:** ***Blastocystis*** **faecal microscopy, XIVC and PCR results before and after triple antibiotic therapy**

**Patient identity**	**Time**	**Faecal microscopy**		**PCR-WONG**		**PCR-ST1 specific**		**PCR-ST3 specific**		**PCR-ST4**		**Clearance**
		**Faeces**	**XIVC**	**Faeces**	**XIVC**	**Faeces**	**XIVC**	**Faeces**	**XIVC**	**Faeces**	**XIVC**	
1	**Baseline**	**+**	**+**	ST4*	ST6*	**Negative**		**Negative**	ST3*	**Negative**	**ST4**	
	**Day 15**	**Negative**	**Negative**	ST4*	**Negative**	**Negative**	**Negative**	**ST3**	**Negative**	**Negative**	**Negative**	**Yes**
	**Week 6**	**Negative**	**Negative**	**Negative**	**Negative**							**Yes**
2	Baseline	+	+	ST7*	Negative	Negative		Negative		Negative		
	Day 15	+	+	+	Negative							No
	Week 6	+	+	ST7*	Negative	Negative		Negative		Negative		No
3	**Baseline**	**+**		ST1*		**ST1**		**Negative**		**Negative**		
	**Day 15**	**+**	**+**	**+**		**Negative**		**Negative**		**Negative**		**No**
	**Week 6**	**+**	**+**	**Negative**	**Negative**							**No**
4	Baseline	+	+	Negative	ST3*	Negative	Negative	Negative	ST3	Negative	Negative	
	Day 15	Negative	Negative	Negative	Negative							Yes
	Week 6	Negative	Negative	Negative	Negative							Yes
5	**Baseline**	**+**	**+**	ST4*	**Negative**	**Negative**		ST3*		**ST4**		
	**Day 15**	**Negative**	**+**	**Negative**	ST6*	**Negative**		ST3*		**Negative**		**No**
	**Week 6**	**Negative**	**+**	ST4*	**Negative**	**Negative**		ST3*		**ST4**		**No**
6	Baseline	+	+	ST4*	Negative	Negative		Negative		ST4		
	Day 15	Negative	Negative	ST1*	Negative	Negative		Negative		Negative		Yes
	Week 6	Negative	Negative	Negative	Negative							Yes
7	**Baseline**	**+**	**Negative**	**Negative**	ST4*	**Negative**		ST3*		**Negative**		
	**Day 15**	**Negative**	**Negative**	**Negative**	**Negative**							**Yes**
	**Week 6**	**Negative**	**Negative**	**Negative**	**Negative**							**Yes**
8	Baseline	+	+	ST8*	ST8*	Negative	Negative	Negative	Negative	Negative	Negative	
	Day 15	+		ST8*		Negative		Negative		Negative		No
	Week 6	+	+	ST8*	ST8*	Negative	Negative	Negative	Negative	Negative	Negative	No
**9**	**Baseline**	**+**	**+**	**Negative**	ST3*		**Negative**		**ST3**		**Negative**	
	**Day 15**	**Negative**		**Negative**								**Yes**
	**Week 6**	**Negative**	**Negative**	**Negative**	**Negative**							**Yes**
10	Baseline	+	+	Negative	ST3*		Negative		ST3		Negative	
	Day 15	+	+	ST3*	Negative	Negative		ST3		Negative		No
	Week 6	Negative	Negative	Negative	Negative							Yes

XIVC: xenic invitro culture.

Polymerase chain reaction PCR.

*Sequenced.

### Clearance and clinical follow up

Overall 60% of patients cleared the organism from their faeces at 6 weeks. Three of these patients had had previous courses of antibiotics. The patient’s were reviewed clinically and their patient diary scored. The average score for the baseline week and week 4 following TAB therapy was calculated taking the weekly mean of the number of daily bowel actions, consistency of stool and general wellbeing. The final clinical score was calculated as the sum of improvement in frequency of bowel habit, consistency and wellbeing from baseline to week four after antibiotic therapy (see Additional file 1). Six patients reported that they felt clinically improved and this correlated with a positive change of greater than 1.0 diary score from baseline to six weeks. However, of these six clinically improved patients only three demonstrated faecal clearance of the organism. In contrast, four patients did not report clinical improvement although two of these patients demonstrated faecal clearance of *Blastocystis* spp. This data was analysed to assess whether clinical improvement or *Blastocystis* clearance had any relationship to age of patient, duration of symptoms, naïve therapy status, original *Blastocystis* faecal load, the particular *Blastocystis* subtype, serum IgA level or clinical outcome. Significantly higher rates of *Blastocystis* spp. clearance were observed if the patient had been symptomatic for less than a year (Mann–Whitney, p = 0.032, 95% confidence), but no other associations were found (Table 2).

**Table 2:** Clinical characteristics and outcome after triple antibiotic therapy

**Patient**	**Age yrs**	**Duration of symptoms (years)**	**Previous AB therapy**	***Blastocystis*** **load in baseline faeces**	***Blastocystis*** **ST’s**	**SerumIgA g/L**	**Diary score**	**Clinical score > =1 point**	**Clearance at 6 weeks**
1	35	4	Yes (F,O)	2+	3,4,6	1.51	+4.1	Yes	**Yes**
2	59	5	No	2+	7	1.12 (L)	+2.4	Yes	No
3	28	1.5	Yes (F)	1+	1	2.1	−0.3	No	No
4	35	0.25	Yes (F,O)	1+	3	2.2	+0.2	No	**Yes**
5	51	1.5	Yes (F)	1+	3,4,6	2.82	+2.0	Yes	No
6	58	0.5	No	2+	1,4	1.03 (L)	+1.1	Yes	**Yes**
7	73	0.75	No	1+	3,4	1.22	+4.5	Yes	**Yes**
8	49	10	Yes (F,O)	3+	8	1.94	+1.3	Yes	No
9	63	1	No	1+	3	0.75 (L)	−0.8	No	**Yes**
10	36	1	Yes (F)	2+	3	0.98 (L)	−0.6	No	**Yes**

F = Flagyl.

O = other antibiotics (metronidazole, nitazoxanide, norfloxacillin).

L = low, <1.24 g/L, level defined by laboratory.

## Discussion

Successful faecal eradication of *Blastocystis* spp.occurred in 60% of patients at the end of this study following fourteen days of triple antibiotic therapy using diloxanide furoate, trimethoprim/sulfamethoxazole and secnidazole (TAB). Our previous study [22] used metronidazole as a monotherapy and achieved 0% successful faecal eradication, suggesting that this TAB regimen may be useful to treat *Blastocystis* infection. Although six patients in our study had already received at least one course of metronidazole therapy previously, three of these patients proceeded to clear the organism after TAB therapy indicating that triple therapy is still a worthwhile alternative in this group. One recent study has reported 100% failure to clear in five patients with *Blastocystis* ST3 infection treated with TAB combination [26]. However, these five patients had faecal specimens retested 12 months following the TAB therapy and it was not possible to exclude reinfection in this group. Triple antibiotic therapy has been shown to be useful in treating infecting organisms such as *Helicobacter pylori* and *Mycobacterium spp*. and it may be necessary to explore combinations of antibiotics and methods of delivery to further improve the clearance rate of *Blastocystis* infection.

Widely varying rates of eradication (0-100%) of *Blastocystis* using a variety of antibiotics including ketoconazole, ornidazole, tinidazole, rifaximin and paromomycin have been reported [21] and some of this apparent inconsistency may be due to using insensitive faecal smears as a sole means of diagnosis. Diagnostic methods such as faecal XIVC or PCR have been reported to increase accuracy by 30-50% for detection of *Blastocystis* spp. [10]. Metronidazole resistance has also been reported [27] and differences in antimicrobial sensitivities to metronidazole and emetine have been demonstrated between axenic ST4 and ST7 cultures [28]. Resistance may therefore contribute to regional variation in eradication rates. A number of antimicrobials have been tested for activity against *Blastocystis* spp. *in vitro*, in both axenic and xenic conditions [29]–[31] and this data is a useful starting point for choice of antimicrobial. The *Blastocystis* organism appears to establish in the anaerobic environment of the human ileum and caecum, thrives in the presence of bacteria, and only in extremely rare circumstances is invasive to the gastrointestinal mucosa [1]. An intrinsic difficulty in treating *Blastocystis* spp. may be delivering the appropriate drugs to the intraluminal site of infection or understanding how to alter the faecal microbiota to produce an environment that is unsupportive or hostile to the parasite.

Secnidazole, similar to metronidazole, is a 5-imidazole drug substituted at position 1 and has been shown to have significant (although 50% less than metronidazole) inhibitory activity against *Blastocystis* spp. *in vitro* tested on axenic cultures [31]. All nitroimidazole drugs are rapidly absorbed from the small bowel, metabolised in the liver and excreted primarily in the urine. They exert their antimicrobial action in an anaerobic intracellular environment after reduction to a toxic metabolite that disrupts DNA linkage [32]. Metronidazole is the most common empiric therapy used against *Blastocystis*[33] and as many patients that present for therapy have already failed metronidazole we felt it was useful to use a different imidazole particularly as resistance to metronidazole has been shown not to extend across the entire class of 1-sustituted 5-imidazoles [28]. The pharmacodynamics of secnidazole are not as well defined as those of metronidazole but it is known that secnidazole has reduced excretion of parent drug and metabolites in the urine (50% compared to 90% with metronidazole) and therefore may have increased faecal excretion. It also has more than double the excretion half-life of metronidazole at 29 hours [28]. These pharmacologic properties may be an advantage in treating *Blastocystis*.

Trimethoprim/sulfamethoxazole duotherapy is often proposed as second line therapy for *Blastocystis* spp. infection and eradication rates of 22-100% have been reported for this combination therapy [21]. These two drugs act synergistically to interfere with the production of dihydrofolate reductase that is required for purine and including DNA synthesis. *Blastocystis* ST7 does not take up ^H^thymidine [18],[31], is likely to synthesize purines de novo, and likely to have a dihydrofolate reductase that can be targeted. Trimethoprim is rapidly absorbed from the small bowel [34] is lipophilic with wide tissue distribution and 80% is excreted in the urine unchanged. Trimethoprim therapy alone has been shown to have inhibitory activity similar to secnidazole *in vitro*[31]. Sulfamethoxazole similarly is rapidly absorbed in the small bowel [34] metabolised in the liver, and over 90% excreted in the urine. They both have a half-life of around 10 hours. Although sulfamethoxazole alone was found to be non-inhibitory *in vitro*[28] a combination of trimethoprim:sulfamethoxazole (in the ratio of 1:5, Bactrim®) was found to be much less effective at inhibiting *Blastocystis* growth in axenic cultures than a trimethoprim/sulfamethoxazole 1:2 ratio which may indicate that the synergistic effect of the sulfamethoxazole plays an important role in directly inhibiting *Blastocystis* spp. growth. Enteric coating this duotherapy compounded in a 1:2 ratio may be a useful therapeutic avenue to explore.

An alternative therapeutic approach would be to use an antimicrobial drug that has already been described to have intraluminal amoebocidal properties. A number of these drugs tested *in vitro* such as emetine, furazolidone, nitazoxanide, mefloquine, quinicrine, quinine, iodoquinol, clioquinol (Entero-Vioform®) and chloroquine showed moderate to high inhibition on *Blastocystis* cell growth [28]–[31]. Emetine and clioquinol therapy have serious adverse reactions reported and are not used currently in clinical practice. Other intraluminal drugs such as diloxanide furoate, paromomycin, doxycycline and pyrimethamine showed no inhibitory effect on *Blastocystis*[29],[30]. Paromomycin is a relatively safe, poorly absorbed aminoglycoside with activity against not only gram-negative but many gram-positive bacteria, including *Mycobacterium tuberculosis,* and protozoa such as *Leishmania* spp. [35]. Interestingly two recent studies have reported high *Blastocystis* clearance rates (77-100%) [26],[36] using paromomycin and if confirmed this would suggest the broad spectrum aminoglycoside is achieving success by an indirect effect on the faecal microbiota. The largest study [36] retrospectively analysed faecal microscopy results on 52 symptomatic *Blastocystis* carriers treated with paromomycin alone and clearance rates were estimated to be 77%. Rifaximin is an oral broad-spectrum antibiotic derived from rifamycin that is not absorbed systemically (97% excreted in the faeces) [37]. It has been used successfully to treat hepatic encephalopathy, travellers diarrhoea [37] and non-constipated IBS patients [38]. Only one case report of its successful use in *Blastocystis* infection has been published [39]. Diloxanide furoate was chosen for our study as it had a good safety profile, the drug remains intraluminal until it is hydrolysed to diloxanide, absorbed and subsequently excreted in the urine. It is known to effectively clear intestinal amoebiasis although the mechanism of action is not known [40]. It was not possible to assess whether the addition of diloxanide furoate increased the efficacy of the regimen in this study. The addition of an intraluminal antimicrobial to a therapeutic regimen seems logical and paromomycin may be the most appropriate choice to add to a regimen at the moment. Unfortunately monotherapy is usually a cheaper option (the cost of 14 days of appropriate doses of metronidazole, trimethoprim/sulfamethoxazole, diloxanide furoate, paromomycin, secnidazole and rifaximin being AUS $24, $24, $55, $70, $80, $80 respectively) and the cost of TAB rising to AUS$159. If an effective drug combination could be identified it is likely the costs of a combination therapy would reduce.

This small pilot study was not designed to interrogate specific variables that might be relevant to *Blastocystis* spp. symptoms, clearance or clinical outcome. The literature is inconsistent but some studies have suggested that particular subtypes, namely ST 1, 3 and 4 of *Blastocystis* may be pathogenic [1]. In contrast, and in accord with recent studies [22],[26] our patients demonstrated a diverse range of subtypes and no association was seen between subtype and faecal clearance or resolution of symptoms. Although previous studies have reported mixed infection rates of 5-30% [22],[41] almost half of the patients (40%) in this study demonstrated mixed infections. The use of ST specific primers and XIVC techniques that allow ST’s present in small numbers to grow to detectable numbers are factors likely to increase identification of mixed infections.

It is well recognised that many *Blastocystis* carriers are healthy. Much research has been directed towards identifying genetic characteristics specific to the parasite, such as ST, that may indicate pathogenicity. Some studies have investigated host factors that may allow *Blastocystis spp.* to cause clinical symptoms. Epidemiological studies conducted in patients with compromised immune function (HIV carriage, chronic renal failure, haematological malignancies with immunosuppressant therapy [42],[43]) have shown increased rates of *Blastocystis* carriage but no clear consensus has emerged linking carriage to symptoms. IBS [6] and *Blastocystis* infection [14] have both been linked to changes in inflammatory cytokine expression. The prevalence of single nucleotide gene polymorphisms present in IL-8 (pro-inflammatory) and IL-10 (anti-inflammatory) cytokines is reported to be significantly increased not only in IBS patients but also in IBS patients positive for *Blastocystis* infection [44] suggesting that factors regulating the immune response in the host are important in expression of clinical disease.

IgA is also an important part of host mucosal immunity. *Blastocystis* proteases have been reported to cleave human secretory IgA [16]. Interestingly 40% of our group of IBS-D *Blastocystis* had low serum IgA and this was much higher than the 1.4% (1 in 19) recorded in our healthy adult volunteers negative for *Blastocystis* spp. (unpublished observations). The levels of serum IgA recorded in our patients are compatible with partial selective IgA deficiency but did not reach <50% of the lower limit of normal defined as selective IgA deficiency. Selective IgA deficiency (prevalence of 0.17% in Australia) is usually asymptomatic, but may be associated with an increased risk of respiratory, urinary and gastrointestinal (including giardiasis) sepsis, and allergic and autoimmune diseases [45]. There was no apparent association between IgA levels and subsequent clearance. Serum IgA levels recorded in children are generally lower than adults [46] and if IgA levels influence the risk of carriage of *Blastocystis* then children may be more susceptible.

In this paper we describe successful clearance of the *Blastocystis* organism from faeces after testing at day 15 and week 4 after antibiotics. *Blastocystis* organisms may be excreted irregularly [47] so it was useful to have had two clear faecal specimens 4 weeks apart to confirm faecal clearance. We used three methods of detecting *Blastocystis* in the stool, taking care to include XIVC and PCR amplification of *Blastocystis* DNA as the latter two methods have approximately a 90% sensitivity detection rate compared with only around 50% for microscopy alone [48]. Nevertheless false negative results may occur in a small percent of cases, where complete clearance of *Blastocystis spp.* in the ileum may not have occurred. It would be useful to be able to distinguish symptomatic and asymptomatic patients positive for *Blastocystis* faecal carriage. Although *Blastocystis* spp. are non-invasive organisms serum antibodies against *Blastocystis* spp. have been reported in humans infected with the parasite [49]. The presence of antibodies often only signifies exposure to that *Blastocystis* antigen some time in the past. However one study has reported that an antibody reacting to a 29 kDa protein, thought to be a cysteine protease present on the plasma membrane of *Blastocystis* spp., is more commonly seen in symptomatic patients [50]. If confirmed this finding and similar approaches may allow us to utilise the expression of host response to infection to determine need for therapy.

The only variable that was found to be significant was duration of GIT symptoms and eventual clearance of the parasite. If we assume that the GIT symptoms were related to the *Blastocystis* carriage this finding may suggest that *Blastocystis* infection that persists in the host for years may be more difficult to eradicate, particularly as spontaneous clearance has been reported to occur in only 19% of symptomatic patients over 12 months [36].

The patients in this study were a careful selection of D-IBS patients with no other identifiable enteric pathogen or other cause for symptoms. Nevertheless only 50% of patients who reported clinical improvement had cleared the organism. Perhaps reducing parasite load was enough to achieve clinical improvement in these patients. The two patients who reported dramatic clinical improvement (>4 points) both cleared the organism. In contrast 50% of the patients who did not report clinical improvement cleared the organism, suggesting that *Blastocystis* carriage was not the cause or not the only cause of their D-IBS. IBS is a heterogenous clinical condition and many diseases masquerade as IBS. Over time definitive specific diagnostic tests or therapies that allow us to discriminate other causes of IBS may become available. Although *Giardia duodenalis* was identified microscopically in the 1700’s acceptance of its pathogenicity only occurred in the last forty years when effective therapy with imidazoles (>90% successful eradication) demonstrated clinical improvement [51]. Although 60% patients cleared in this study clinical cause and effect will be able to be more easily evaluated when we can more reliably and effectively eradicate *Blastocystis* spp.

## Conclusions

Successful faecal eradication of *Blastocystis* spp. occurred in 60% of D-IBS patients at the end of this study following fourteen days of triple antibiotic therapy using diloxanide furoate, trimethoprim/sulfamethoxazole and secnidazole (TAB). Metronidazole monotherapy achieved 0% successful eradication in our previous study and as some isolates of *Blastocystis* spp. have also been shown to be resistant to metronidazole, this drug should no longer be recommended as first line therapy. Surprisingly paromomycin, a drug that exhibits little inhibitory activity *in vitro*, has been reported to have eradication rates above 75%. If these results are confirmed in future studies using sensitive diagnostic markers, paromomycin may prove to be a useful basis for a double or triple antibiotic regimen. Predictable successful eradication (>90%) of *Blastocystis* spp. will expedite investigation of the clinical significance of *Blastocystis* infection. No meaningful analysis of clearance and symptoms was possible in this trial but 40% *Blastocystis* patients demonstrated low serum IgA levels. It may be worth focusing future studies on investigating immune profiles in the host and including host IgA levels.

## Competing interests

The authors declare they have no competing interests.

## Author’s contributions

RN carried out the clinical investigations, microbiology and molecular genetic studies, sequence alignment, statistical analysis and drafted the manuscript. RN, HBO and RT conceived the study design, participated in the design and coordination and helped draft the manuscript. All authors read and approved the final manuscript.

## Additional file

## Supplementary Material

Additional file 1: **Clinical Diary of** ***Blastocystis*** **patients during study period.** Click here for file
