# Supplementary material for: Clinical pilot study: efficacy of triple antibiotic therapy in Blastocystis positive irritable bowel syndrome patients
Source: Gut Pathog. 2014 Aug 20;6:34. doi: 10.1186/s13099-014-0034-0 (PMC4209510; doi:10.1186/s13099-014-0034-0)
Supplement: Additional file 1: — Clinical Diary of Blastocystis patients during study period. [file s13099-014-0034-0-S1.doc]

|  | **1** | | | **2** | | | **3** | | | **4** | | | **5** | | | **6** | | | **7** | | |  |
| --- | --- | --- | --- | --- | --- | --- | --- | --- | --- | --- | --- | --- | --- | --- | --- | --- | --- | --- | --- | --- | --- | --- |
| **Time/**  **Patient** | **Baseline** | | | **AB Week 1** | | | **AB Week 2** | | | **After AB**  **Week 1** | | | **After AB**  **Week 2** | | | **After AB**  **Week 3** | | | **After AB**  **Week 4** | | | **Clinical Score** |
|  | ***F*** | ***C*** | ***WB*** | ***F*** | ***C*** | ***WB*** | ***F*** | ***C*** | ***WB*** | ***F*** | ***C*** | ***WB*** | ***F*** | ***C*** | ***WB*** | ***F*** | ***C*** | ***WB*** | ***F*** | ***C*** | ***WB*** |  |
| **1** | 2.6 | 3.4 | 6 | 2.9 | 3.6 | 5.6 | 3.3 | 3.9 | 7.4 | 2.4 | 3.3 | 7.4 | 2.7 | 3.0 | 7.4 | 2.4 | 3.4 | 7.9 | 2.1 | 3 | 9 | +4.1 |
| **2** | 1.5 | 3 | 5.1 | 1.3 | 2.7 | 5.6 | 1.1 | 2.6 | 6.9 | 1.3 | 2.9 | 6.1 | 1.4 | 3.0 | 7.0 | 1.3 | 2.4 | 7.0 | 0.9 | 2.8 | 6.6 | +2.4 |
| **3** | 3.0 | 3.3 | 5.6 | 2.1 | 2.7 | 4.4 | 3.7 | 3.0 | 4.0 | 3.3 | 3.1 | 4.0 | 2.4 | 3.0 | 4.3 | 2.3 | 2.7 | 5.0 | 2.7 | 3.3 | 5.0 | -0.3 |
| **4** | 2.6 | 3.5 | 7.2 | 2.6 | 3.1 | 6.9 | 3.1 | 3.4 | 6.8 | 3.2 | 3.5 | 6.9 | 2.8 | 3.6 | 6.9 | 2.8 | 3.5 | 7.0 | 2.5 | 3.6 | 7.4 | +0.2 |
| **5** | 1.6 | 3.4 | 7.1 | 1.3 | 2.2 | 8.0 | 1.0 | 2.6 | 8.0 | 1.3 | 2.3 | 8.0 | 1.4 | 3.1 | 8.0 | 1.3 | 3.0 | 8.1 | 1.0 | 3.0 | 8.1 | +2.0 |
| **6** | 1.3 | 3.4 | 7.1 | 1.8 | 3.3 | 7.1 | 1.4 | 3.7 | 6.9 | 1.3 | 3.5 | 7.5 | 1.3 | 3.5 | 7.4 | 1.3 | 3.2 | 7.5 | 1.2 | 3.1 | 7.8 | +1.1 |
| **7** | 4.4 | 4.0 | 5.9 | 2.0 | 2.7 | 6.0 | 1.3 | 2.5 | 6.0 | 2.1 | 3.0 | 6.9 | 2.0 | 2.9 | 6.6 | 2.0 | 2.9 | 6.7 | 2.2 | 2.8 | 7.0 | +4.5 |
| **8** | 2.6 | 3.0 | 7.0 | 3.0 | 3.1 | 6.7 | 2.7 | 3.3 | 7.3 | 1.3 | 3.0 | 7.6 | 1.4 | 3.0 | 7.0 | 1.6 | 3.4 | 7.1 | 1.6 | 3.1 | 7.4 | +1.3 |
| **9** | 1.6 | 2.0 | 9.0 | 2.0 | 3.0 | 7.8 | 4.4 | 3.0 | 7.3 | 4.4 | 3.0 | 8.3 | 2.0 | 3.0 | 8.8 | 1.0 | 2.2 | 9.0 | 2.0 | 2.2 | 8.8 | -0.8 |
| **10** | 2.0 | 2.4 | 6.7 | 2.4 | 3.2 | 3.6 | 2.0 | 2.6 | 6.4 | 2.1 | 2.1 | 4.1 | 1.9 | 2.2 | 5.2 | 1.2 | 2.5 | 6.5 | 1.6 | 2.7 | 6.0 | -0.6 |

**Supplementary File 1: Clinical Diary**

F=mean bowel frequency over 7 days C=mean consistency over 7 days WB=mean well being score over 7 days

F=number of bowel movements per 24 hours

C=consistency of bowel action scored: 1= very hard 2=hard 3=formed 4=loose 5=watery

WB= feeling of wellbeing assessed daily scored 1=feels terrible 10=best ever

Final Clinical score = Improvement in stool frequency (1-7) + improvement in stool consistency (1-7) +improvement in wellbeing (7-1)
